# Comparison among the available stone treatment techniques from the first European Association of Urology Section of Urolithiasis (EULIS) Survey: Do we have a Queen?

**DOI:** 10.1371/journal.pone.0205159

**Published:** 2018-11-02

**Authors:** Stefano Paolo Zanetti, Michele Talso, Franco Palmisano, Fabrizio Longo, Andrea Gallioli, Matteo Fontana, Elisa De Lorenzis, Gianluca Sampogna, Luca Boeri, Giancarlo Albo, Alberto Trinchieri, Emanuele Montanari

**Affiliations:** 1 Department of Urology, IRCCS Ca’ Granda Ospedale Maggiore Policlinico, Università degli Studi di Milano, Italy; 2 Department of Urology, ASST Vimercate Hospital (MB), Italy; 3 Department of Urology, Presidio Ospedaliero Alessandro Manzoni, Lecco, Italy; University of Mississippi Medical Center, UNITED STATES

## Abstract

**Purpose:**

The miniaturization of instruments has had an impact on stone management. The aims of this study were to highlight surgeon preferences among Retrograde Intra Renal Surgery (RIRS), Regular, Mini-, UltraMini- and Micro- Percutaneous Nephrolithotomy (PCNL) for urolithiasis and to compare the effectiveness and safety of these techniques in a real-life setting.

**Methods:**

A 12-item survey regarding endourological techniques was conducted through *Survey Monkey* among attendees of the 2013 European Association of Urology Section of Urolithiasis meeting. We asked responders to share data from the last 5 cases they performed for each technique. Procedures were stratified according to stone size and the centres’ surgical volume. Techniques were compared in terms of effectiveness and safety. Analyses were performed on the overall group and a subgroup of 1–2 cm stones.

**Results:**

We collected data from a total of 420 procedures by 30, out of 78, urologists who received the survey (response rate 38%): 140 RIRS, 141 Regular-PCNL (>20 Ch), 67 Mini-PCNL (14–20 Ch), 28 UltraMini-PCNL (11–13 Ch) and 44 Micro-PCNL (4,8–8 Ch). Techniques choice was influenced by stone size and the centre’s surgical volume. Effectiveness and safety outcomes were influenced by stone size, independently of the technique. The stone-free rate was significantly lower in Micro-PCNL compared to Regular-PCNL. This was not confirmed for 1–2 cm stones. All techniques presented a lower complication rate than Regular-PCNL, with Mini-PCNL being the most protective technique compared to Regular-PCNL.

**Conclusions:**

Stone size seems to drive treatment choice. Miniaturized PCNL techniques are widely employed for 1–2 cm stones, in particular in higher surgical volume centres. Mini-PCNL and RIRS are growing in popularity for stones > 2 cm. Mini-PCNL seems to be a good compromise, being the most effective and safe procedure among PCNL techniques. RIRS is characterized by satisfactory stone-free and low complication rates.

## Introduction

Nephrolithiasis is a common disease with a high worldwide prevalence that ranges from 7 to 13% in North America, 5–9% in Europe, and 1–5% in Asia [1]. Geography, diet, fluid intake, genetics, climate, age, and occupation are important factors that can affect the incidence of this disease. Kidney stone management is expensive and surgery is often the gold standard treatment. The European Association of Urology Guidelines define External Shock Wave Lithotripsy (ESWL) or Endourology as the first-choice treatments for 1–2 cm kidney stones, where the term ‘Endourology’ encompasses all Percutaneous Nephrolithotomy (PCNL) and Ureterorenoscopy (URS) interventions. For stones bigger than 2 cm the gold standard is PCNL treatment [2]. Nowadays, the panorama of PCNL instrumentation is extremely wide and includes Micro-PCNL with 4,8 Ch, UltraMini-PCNL (UMP) with 11–13 Ch, Mini-PCNL with 14–20 Ch, Regular-PCNL with > 20 Ch access diameters. The miniaturization of instruments has impacted indications, as smaller tracts theoretically reduce complications such as blood loss and renal parenchyma injury [3–4]. Retrograde Intra Renal Surgery (RIRS) is included among the first line treatments for kidney stones between 1 and 2 cm, but could be proposed as a viable alternative therapy to PCNL for stones larger than 2 cm in special groups of high-risk patients (e.g. those with bleeding disorders, obesity, renal congenital abnormalities, or solitary kidney) [5]. The guidelines consider each of these techniques but don’t provide specific indications for the different sized PCNL techniques and do not univocally define the best treatment option for kidney stones, especially for calculi between 1 and 2 cm.

### Purpose

The aim of our study is to highlight surgeons’ preferences among RIRS, Regular, Mini-, UltraMini- and Micro-PCNL in stone treatment in a real-life setting, based on a Survey approved by the European Association of Urology Section of Urolithiasis (EULIS).

Moreover, we compared the effectiveness and safety of these techniques in stone treatment, with a particular interest on the grey zone of 1–2 cm stones.

## Materials and methods

We performed a Survey approved by the EULIS board in Cape Town on December 2014 regarding the employment of RIRS and regular and small sized PCNL among physicians who attended the Copenhagen EULIS meeting in 2013. The project was carried out in three steps using *Survey Monkey* and took place from February 2015 (collection of the first and second-step results) to May 2015 (third step). This article is focused on the results from the third step. An introductory email was sent to all participants, describing the study and providing the link to the online questionnaire. Participation was voluntary. The first two steps aimed to delineate the departments’ profiles in terms of instrument availability and performed techniques, and to collect experts’ personal opinions on RIRS and regular and small size PCNL.

In the *third step* responders were asked to report the number of procedures performed during the previous year and to share information regarding the last 5 cases treated in their centres with each of the techniques under study for which they had at least one-month of follow-up. They were asked to report data concerning different pre-, intra- and post-operative items. Twelve of these items were common for all procedures: 5 were related to patient, stone or operation characteristics (i.e. age, sex, comorbidities, stone size and exit strategy), 2 were considered *effectiveness* outcomes (i.e. *one month stone-free status* and *need for retreatment)* and 5 were *safety* outcomes (i.e. *operation time*, *Hb drop*, *need for transfusions*, *hospital stay duration* and *complications* according to the Clavien/Dindo scale). Stone size was defined as the maximum stone diameter. In cases of multiple stones the cumulative maximum stone diameter was used. Stones were categorized in three groups according to their size (i.e. < 1 cm, 1–2 cm, > 2 cm). Operation time was dichotomized by surgeries lasting ≤ 60 minutes and those lasting longer. Stone free status was defined as the total absence of residual fragments at the 1-month follow-up evaluation. The imaging modality used to determine stone presence/absence was dependent on each centre’s local protocol. The questionnaires we sent to collect data on RIRS and PCNL procedures are available in the supporting information files (S1 and S2 Figs, respectively).

Procedures were stratified according to stone size and each centre’s surgical volume (>/<50 PCNL procedures performed during the previous year).

We explored whether or not effectiveness and safety outcomes differed in relation to stone size, using the Chi-squared test for qualitative outcomes and linear regression analysis for quantitative outcomes, previously log transformed if appropriate (i.e. hospital stay and haemoglobin drop). Effectiveness and safety outcomes for the different techniques were compared using multiple regression models (logistic for categorical outcomes and linear for quantitative outcomes) after correction for stone size, age and gender. The same analyses were also performed on a subgroup of 1–2 cm stones in order to analyse a more homogeneous group in terms of number of procedures. Statistical analyses were performed with the Stata 10 package considering statistical significance for P values < 0.05.

This study protocol was reviewed and approved by the ethics committee of the University of Milan and all clinical investigations were conducted according to the principles expressed in the Declaration of Helsinki. Every patient (parents if minor or guardians if incompetent) signed a written informed consent for his/her clinical information to be used and shared for research purposes.

## Results

We sent the first and second step questionnaires to 360 people and obtained a 24% response rate (88 responders). Seventy-eight physicians agreed to receive the third step questionnaire and 38% of these (30 responders) shared data from their recent procedures using each technique. Not all responders shared 5 cases for each technique. We collected a total of 420 procedures: 140 RIRS from 28 centres, 141 Regular PCNL (>20 Ch) from 27 centres, 67 Mini-PCNL (14–20 Ch) from 15 centres, 28 UMP (11–13 Ch) from 7 centres and 44 Micro-PCNL (4,8–8 Ch) from 8 centres. The 420 procedures were performed on an equal number of patients. The sample population had median age of 51 years (2–89) with a slight prevalence of males (57.86%). The techniques performed by the single centres were stratified according to their surgical volume as shown in Table 1.

**Table 1:** Stratification of the procedures according to centres’ surgical volume.

Centres surgical volume	Centres n.	Centres performing Regular-PCNL n. (%)*	Centres performing Mini-PCNL n. (%)*	Centres performing UMP n. (%)*	Centres performing Micro-PCNL n. (%)*
**< 50 PCNL/year**	17	15/17 (88%)	6/17 (35%)	2/17 (12%)	4/17 (23%)
**> 50 PCNL/year**	13	12/13 (92%)	9/13 (69%)	5/13 (38%)	4/13 (31%)

*Numbers and percentages of centres which performed at least 1 procedure are reported for each technique in the 2 groups.

Responders reported statistically significant differences in the chosen technique in relation to stone size (Table 2).

**Table 2:** Comparison of the chosen techniques according to stone size. Chi-squared test results are reported.

Stone size	Regular PCNL n. (%)	Mini-PCNL n. (%)	UMP n. (%)	Micro-PCNL n. (%)	RIRS n. (%)	Tot n. (%)	P-value
**≤ 1 cm**	3 (5%)	1 (2%)	2 (3%)	7 (11%)	49 (79%)	62 (100%)	<0.0001
**1–2 cm**	26 (15%)	30 (17%)	24 (14%)	29 (17%)	63 (37%)	172 (100%)	
**> 2 cm**	112 (60%)	36 (20%)	2 (1%)	8 (4%)	28 (15%)	186 (100%)	

As shown in Table 3, the effectiveness and safety outcomes were also influenced by stone size, independently of the technique used.

**Table 3:** Comparison of effectiveness and safety outcomes among stones of different sizes. For categorical variables the number of outcomes, percentages and Chi-squared test results are reported, while for quantitative outcomes (*) medians, ranges and linear regression results are reported.

Outcome	≤ 1 cm	1–2 cm	> 2 cm	Total	P value
**One month stone free n. (%)**	58 (93.55%)	141 (82.46%)	127 (68.28%)	326 (77.80%)	**< 0.0001**
**Second procedure needed n. (%)**	2 (3.23%)	12 (7.06%)	35 (18.82%)	49 (11.72%)	**< 0.0001**
**Operative time (> 60 min) n. (%)**	53 (85.48%)	91 (55.15%)	57 (30.65%)	201 (48.67%)	**< 0.0001**
Hb drop* Median (interquartile range)	0 (0–4.2)	0.4 (0–3.9)	1.2 (0–6.8)	0.6 (0–6.8)	Coef. 0.46 (CI 0.29–0.63) **< 0.0001**
**Transfusion needed n. (%)**	1 (1.64%)	3 (1.78%)	13 (6.99%)	17 (4.09%)	**0.027**
**Clavien/Dindo scale ≤1 n. (%)**	60 (96.77%)	158 (91.86%)	158 (85.41%)	376 (89.74%)	**0.019**
Hospital stay * Median (interquartile range)	2 (0–10)	2 (0–11)	4 (0–30)	3 (0–30)	Coef. 0.36 (CI 0.26–0.46) **< 0.0001**

The results of the multiple regression analysis assessing whether or not effectiveness outcomes are influenced by the chosen technique (on all procedures, N = 420 and on procedures for 1–2 cm stones, N = 172) are reported in Table 4. The stone free rate (SFR) was significantly lower in patients treated with Micro-PCNL compared to Regular PCNL, when considering procedures performed for all stone sizes (OR 0.36; P = 0.019).

**Table 4:** Comparison of effectiveness outcomes across different techniques (Regular-PCNL considered as reference) for all procedures reported by the responders and for procedures performed on 1–2 cm stones. The table presents the Odds Ratio (OR) adjusted for stone size, age, and gender.

Outcome	Regular-PCNL		Mini-PCNL			UltraMini-PCNL			Micro-PCNL			RIRS		
	%	OR (95% CI)	%	OR (95% CI)	P-value	%	OR (95% CI)	P-value	%	OR (95% CI)	P-value	%	OR (95% CI)	P-value
**All procedures (N = 420)**														
**One month stone free**	73.1%	Ref	85.1% (0.78–3.79)	1.72 (0.78–3.79)	NS	82.1%	0.60 (0.19–1.87)	NS	70.5%	0.36 (0.15–0.85)	0.019	80.6%	0.62 (0.32–1.21)	NS
**Second procedure needed**	20%	Ref	3.0%	0.15 (0.03–0.66)	0.012	7.1%	0.69 (0.14–3.39)	NS	4.6%	0.37 (0.08–1.73)	NS	10.8%	1.10 (0.49–2.4)	NS
**Procedures for 1–2 cm stones (N = 172)**														
**One month stone free**	88.5%	Ref	93.3%	1.51 (0.23–10.1)	NS	83.3%	0.61 (0.12–3.20)	NS	69.0%	0.27 (0.06–1.16)	NS	80.3%	0.51 (0.13–1.99)	NS
**Second procedure needed**	12.0%	Ref	0.0%	-	-	4.2%	0.31 (0.03–3.27)	NS	6.9%	0.54 (0.08–3.54)	NS	9.9%	0.81 (0.19–3.54)	NS

The distribution of safety outcomes in relation to the different techniques and the results of the multiple regression analysis exploring how the outcomes were influenced by the chosen technique (on all procedures N = 420 and on procedures for 1–2 cm stones N = 172) are reported in Table 5. All considered techniques (i.e. Mini-PCNL, UMP, Micro-PCNL and RIRS) presented a lower complication rate than Regular-PCNL, with Mini-PCNL being the most protective technique compared to Regular-PCNL. The total number of transfusions reported was 17: fourteen (82.35%) in patients undergoing Regular-PCNL (3 of which for 1–2 cm stones); one (5.88%) in a patient undergoing Mini-PCNL, one (5.88%) in a patient undergoing UMP and one (5.88%) in a patient undergoing RIRS.

**Table 5:** Comparison of safety outcomes across different techniques (Regular-PCNL considered as reference) for all procedures reported by the responders and for procedures performed on 1–2 cm stones. The table presents the Odds Ratio (OR) for binary outcomes and regression coefficients (Coef) for multiple outcomes, adjusted for stone size, age and gender.

Outcome	Regular-PCNL		Mini-PCNL			UltraMini-PCNL			Micro-PCNL			RIRS		
	% or Median with range	(95% CI)	% or Median with range	OR or Coef (95% CI)	P-value	% or Median with range	OR or Coef (95% CI)	P-value	% or Median with range	OR or Coef (95% CI)	P-value	% or Median with range	OR or Coef (95% CI)	P-value
**All procedures (N = 420)**														
**Operative time** **(>60 min)**	58.2%	Ref	57.81%	OR 1.44 (0.75–2.75)	NS	33.3%	OR 1.16 (0.42–3.20)	NS	70.5%	OR 5.98 (2.51–14.24)	<0.001	38.6%	OR 1.61 (0.86–3)	NS
**Complications (Clavien/Dindo Scale)** 0	55.3%	Ref	77.6%	OR 0.35 (0.18–0.67)	0.002	78.6%	OR 0.42 (0.15–1.14)	NS	70.5%	OR 0.59 (0.27–1.25)	NS	69.5%	OR 0.53 (0.30–0.93)	0.027
1	24.8%	Ref	14.9%			10.7%			18.2%			28.4%		
2	14.2%	Ref	6.0%			10.7%			11.4%			0.7%		
3a	4.3%	Ref	0.0%			0.0%			0.0%			1.4%		
3b	1.4%	Ref	0.0%			0.0%			0.0%			0,0%		
4a	0.0%	Ref	1.5%			0.0%			0.0%			0.0%		
**Hb drop**	1.6 (0–6.8)	Ref	1 (0–4)	Coef. -0.33 (-0.62- -0.08)	0.018	0.3 (0–2.5)	Coef. -0.67 (-1.1- -0.21)	0.004	0.4 (0–3)	Coef. -0.69 (-1.1- -0.33)	<0.001	0 (0–4.2)	Coef. -1.15 (-1.5- -0.84)	<0.001
**Hospital stay**	4 (1–30)	Ref	3 (1–24)	Coef. -0.28 (-0.48- -0.079)	0.006	2 (0–8)	Coef. -0.44 (-0.75–0.138)	0.005	2 (1–6)	Coef. -0.80 (-1.02–0.52)	<0.001	2 (0–19)	Coef. -0.51 (-0.71- -0.32)	<0.001
**Procedures for 1–2 cm stones (N = 172)**														
**Operative time** **(>60 min)**	26.92%	Ref	37.04%	OR 1.5 (0.46–4.88)	NS	35.00%	OR 1.66 (0.46–6.11)	NS	75.86%	OR 8.73 (2.57–29.69)	0.001	43.55%	OR 2.09 (0.76–5.74)	NS
**Complications (Clavien/dindo Scale)** 0	34.62%	Ref	83.33%	OR 0.12 (0.04–0.42)	0.001	79.17%	OR 0.19 (0.05–0.64)	0.008	68.97%	OR 0.29 (0.1–0.84)	0.022	72.58%	OR 0.21 (0.08–0.51)	0.001
1	46.15%	Ref	10,0%			12.50%			17.24%			25.81%		
2	15.38%	Ref	6.67%			8.33%			13.79%			0,0%		
3a	3.85%	Ref	0.0%			0.0%			0.0%			1.61%		
**Hb drop**	1.7 (0–3.9)	Ref	0.8 (0–3.1)	Coef. -0.40 (-0.92–0.13)	NS	0.5 (0–2.5)	Coef. -0.70 (-1.3- -0.1)	0.020	0.5 (0–3)	Coef. -0.61 (-1.1- -0.08)	0.025	0 (0–2.7)	Coef. -1.45 (-2.0- -0.9)	<0.001
**Hospital stay**	4 (1–11)	Ref	3 (1–8)	Coef. -0.41 (-0.75- -0.07)	0.020	2 (0–8)	Coef. -0.48 (-0.85- -0.1)	0.013	2 (1–6)	Coef. -0.80 (-1.14- -0.46)	<0.001	2 (0–11)	Coef. -0.60 (-0.90- -0.30)	<0.001

## Discussion

This article is based on the first survey within the EULIS group. The main goal in stone treatment is to achieve the highest effectiveness with the lowest morbidity. Over recent years, many authors have made comparisons among the different available techniques but still no clear and univocal indications have been delineated, and a wide heterogeneity exists in the application of the single procedures [6]. While only a randomized controlled trial (RCT) could completely resolve this issue, our study outlines the real-life clinical practice among EULIS members.

### Techniques application

Our data show that the techniques are employed differently according to stone size. In particular, Regular-PCNL is the most used technique for stones > 2 cm, followed by Mini-PCNL and RIRS. For stones < 1 cm, RIRS is widely applied. In cases of 1–2 cm stones the indication is less univocal with a slight prevalence of PCNL (evenly distributed among the different tract size options) over RIRS. The distribution of the employed techniques is not uniform among the respondent centres. In particular, while RIRS and Regular-PCNL are performed by almost all of the responders, Mini-PCNL is practiced by only half of them, and UMP and Micro-PCNL, as expected, are the least performed techniques. The choice of technique also seems to be related to the centres’ surgical volume. Specifically, centres performing more than 50 PCNL/year more often apply miniaturized PCNL techniques (including Mini-PCNL, UMP and Micro-PCNL) than centres performing less than 50 PCNL/year. This could be explained not only by the more robust surgical experience but also by the availability of mini invasive instruments and the related costs.

### Effectiveness

#### Background

S. Mishra et al. demonstrated that Mini- and Regular-PCNL can achieve equally effective results in terms of SFR for 1–2 cm stones (96% and 100% respectively, P value = 0.49) [7]. K. Wilhelm et al. reported comparable results for UMP and RIRS in 10–35 mm stones (SFR 92% and 96% respectively, P value = 0.561) [8]. Other studies have obtained comparable stone clearance rates with micro-PCNL and RIRS for stones up to 1,5 cm (97.1% and 94.1% respectively, P value = 1.0 by R.B. Sabnis et al.; 83.3% and 86.7% respectively, by Kandemir et al.) [9–10]. A review by De et al. reported that minimally invasive percutaneous procedures, including Mini- and Micro-PCNL, could provide higher stone-free rates than RIRS [11]. Nevertheless, many studies in the literature report SFRs over 90% for RIRS performed for stones larger than 2 cm, and thus consider it an attractive alternative to PCNL [12–13]. Furthermore, Micro-PCNL has been described as an adequate treatment method for peculiar indications such as solitary renal stones with a volume <1 000 mm^3^ and low density (HU) [14].

Therefore, great discrepancies exist in the reported outcomes of the available procedures. Moreover, the imaging modality and the timing of stone clearance assessment are variable in the cited studies, with some authors using X-ray KUB at 1 or 3 months [7, 9], others using endoscopic inspection and immediate postoperative ultrasound or low-dose CT 4–8 weeks after the procedure [8] and still others using non-contrast spiral CT at 3 months [10]. This renders the comparisons between techniques even less reliable.

The lack of consensus on the best follow-up imaging modality was probably reflected in the series we analyzed, in which different local protocols were adopted.

#### Overall analysis

In our series, considering all performed procedures, no significant differences were observed in terms of SFR between Regular-PCNL and Mini-PCNL, UMP and RIRS, even if Mini-PCNL seemed to be more effective than UMP and RIRS according to the higher odds ratio. Moreover, Mini-PCNL was the only technique studied that resulted in a significantly lower need for second procedures. Micro-PCNL, rather, presented a significantly lower SFR than Regular-PCNL; nevertheless, the retreatment rate after this technique didn’t differ in a statistically significant way from the other techniques, suggesting that the clinical outcomes may be satisfactory enough to not require a second operation.

#### 1–2 cm stones group

In the 1–2 cm stone group, no statistically significant differences were observed among any of the techniques with respect to Regular-PCNL. However, the results were similar to those described for the total group, with the best outcomes being observed for Mini-PCNL and the lowest SFR for Micro-PCNL.

### Safety

#### Background

As already stated in the literature, reductions in both haemoglobin loss and hospital stay are obtained by reducing the invasiveness of the procedure [11]; S. Mishra et al. demonstrated that Mini-PCNL for 1–2 cm stones is limited by longer operative times than standard-PCNL but is characterized by significantly less bleeding, shorter hospital stays and a similar safety profile [7].

K. Wilhelm et al., in a comparison of UMP and RIRS for 10–35 mm stones, demonstrated that operating times and hospital stays were significantly longer in the UMP group. No patient in their study required a blood transfusion [8]. R.B. Sabnis et al., after randomizing patients to either RIRS or Micro-PCNL for stones < 1.5 cm, demonstrated that Micro-PCNL was associated with greater haemoglobin loss, increased pain and more analgesic requirements, while RIRS was associated with a higher requirement for JJ stenting [9]. A. Kandemir et al., comparing the same techniques for lower pole stones up to 1.5 cm, found similar complication rates but longer scopy times and hospital stays associated with Micro-PCNL [10].

A.Yamaguchi et al., in their study of 5537 patients who underwent PCNL, observed that bleeding and transfusions tended to increase with an increasing size of the access sheath (18 Ch to 34 Ch) [15].

De et al. found that PCNL is related to higher complication rates, blood loss and longer lengths of stay than RIRS, with no differences in surgical time and secondary procedures. Minimally invasive percutaneous procedures, including Mini- and Micro-PCNL, were also found to be related to added morbidity and longer hospital stays than RIRS for stones <2 cm [11].

#### Overall analysis

In the overall group, our results confirmed that each of the small sized PCNL techniques and RIRS produced less haemoglobin loss and shorter hospital stays than Regular-PCNL. With regard to complications, Mini-PCNL was revealed to be the safest among the PCNL techniques. UMP and Micro-PCNL also appeared to be safe procedures, but didn’t result in statistically significant differences, probably due to the smaller number of patients treated with these techniques in our series. RIRS also, and in line with the literature [9,16], was related to significantly lower complication rates than Regular-PCNL. Operative times were comparable to those of Regular-PCNL for all techniques except Micro-PCNL, which appeared to be the most time-consuming procedure.

#### 1–2 cm stone group

Safety results within the 1–2 cm stone group were similar to those of the overall group. Haemoglobin drop and hospital stay progressively decreased with the reduction of invasiveness. For all techniques, high-grade (Clavien ≥2) complication rates were lower than they were for Regular-PCNL. Micro-PCNL appeared to be the most time-consuming technique in this group as well.

### Limitations

One major limitation of this study, intrinsic to the nature of surveys, is the low response rate. This was probably due to the lack of ready availability in all centres to the quested data.

Another limitation of the present study is represented by its multicentre retrospective design, which may have led to a misestimation of some of the outcomes. In particular, the stone free status was assessed with different imaging modalities in accordance with the individual centres’ local protocols, which were not investigated in our survey. This reduces the comparability of the results. Nevertheless, the retreatment rate could be considered as a suitable surrogate for the effectiveness of the procedures.

One further limitation is the small number of miniaturized PCNL procedures reported. This is due, as explained above, to the infrequent application of these techniques in lower surgical volume centres.

We decided to keep the questionnaire as concise and short as possible to maximize the possibility of obtaining responses, only recording fundamental variables. This may have caused us to overlook some characteristics such as stone location and number of stones that could have been useful for the comparison among techniques.

## Conclusions

Stone size seems to be an important factor driving treatment choice. Miniaturized PCNL techniques are widely applied in the treatment of 1–2 cm stones, in particular in higher surgical volume centres. Mini-PCNL and RIRS are gaining in popularity also in the treatment of stones > 2 cm.

The miniaturization of PCNL appears to result in a safer procedure without compromising effectiveness. In particular Mini-PCNL, with accesses ranging from 14 to 20 Ch, seems to be a good compromise, being the most effective and the safest procedure among the PCNL techniques both in the overall group as well as the 1–2 cm stones group. Micro-PCNL appears to be the most time-consuming technique and probably requires highly selected indications. RIRS continues to be a procedure characterized by satisfactory stone-free rates and low complication rates. Nevertheless, considering the limitations of our survey and its retrospective and multicentre design, we cannot draw firm conclusions on the best technique to treat kidney stones. Future RCTs would be the best option for shedding more light on the subject.

## Supporting information

S1 Fig RIRS questionnaire. (PNG)

S2 Fig PCNL procedures questionnaire. (PNG)
