# Supplementary figures and images for: Comparison among the available stone treatment techniques from the first European Association of Urology Section of Urolithiasis (EULIS) Survey: Do we have a Queen?
Source: PLoS One. 2018 Nov 2;13(11):e0205159. doi: 10.1371/journal.pone.0205159 (PMC6214503; doi:10.1371/journal.pone.0205159)

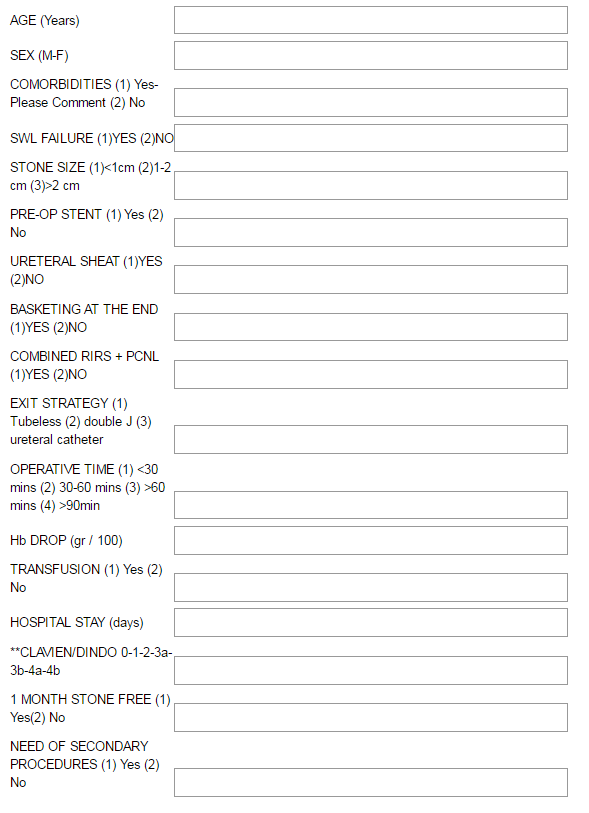

Supplement: S1 Fig — (PNG) [file pone.0205159.s001.png]

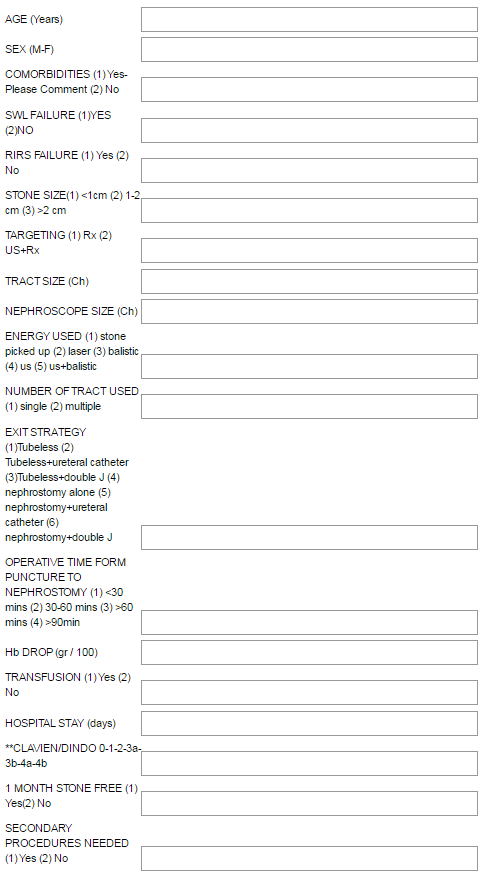

Supplement: S2 Fig — (PNG) [file pone.0205159.s002.png]
